# Hyperammonemia in a septic patient with *Ureaplasma parvum* arthritis: a case report

**DOI:** 10.1186/s12879-022-07953-8

**Published:** 2022-12-22

**Authors:** Xiaohong Pan, Jiekun Xu, Lei Pan, Caihong Wang, Junke Qiu, Xiaqing Huang, Chenxi Yan, Minjie Mao

**Affiliations:** Tuberculosis Care Unit, Hangzhou Chest Hospital Affiliated to Zhejiang University, No. 208 Huancheng East Road, Hangzhou, 310003 Zhejiang China

**Keywords:** Hyperammonemia, *Ureaplasma parvum*, Arthritis, Sepsis

## Abstract

**Background:**

Septic arthritis requires prompt diagnosis and treatments. Rare pathogens should be considered when patients respond poorly to the initial antibiotic treatments. *Ureaplasma parvum* is an opportunistic pathogen that commonly resides in the human urogenital tract. Its infection commonly causes hyperammonemia. Hyperammonemia from *Ureaplasma parvum* septic arthritis has never been reported previously.

**Case presentation:**

A 65-year-old male presented with fever and left lower leg pain and swelling for more than ten days. Septic arthritis and sepsis were considered after laboratory tests and arthrocentesis. However, he responded poorly to the antibiotic treatments, including cefoperazone-sulbactam, imipenem-cilastatin, and linezolid. His mental status deteriorated rapidly with elevated blood ammonia levels with unremarkable liver function test and sonogram examination results. Despite the treatments with lactulose, L-ornithine L-aspartate, mannitol, and hemodialysis therapy to lower his ammonia level, his blood ammonia level remained persistently high. Finally, metagenomic sequencing of the left knee synovial fluid reported *Ureaplasma parvum*, which was considered to contribute to his hyperammonemia.

**Conclusion:**

*Ureaplasma parvum* could cause septic arthritis with hyperammonemia. Genetic tests, such as polymerase chain reaction and next-generation sequencing techniques, could provide a sensitive and fast diagnosis of *Ureaplasma parvum*.

## Background

Septic arthritis requires prompt diagnosis and appropriate treatments to save the affected joints. If patients respond poorly to antibiotic treatments, further exploration of the pathogen should be performed. Here, we reported a septic arthritis patient with hyperammonemia. *Ureaplasma parvum* was finally identified in the left knee synovial fluid and was considered to contribute to hyperammonemia. Hyperammonemia refers to a high blood ammonia level [1]. *Ureaplasma parvum* is a rare cause to induce hyperammonemia [2]. *Ureaplasma parvum* is an opportunistic pathogen that commonly resides in the human urogenital tract [3]. Septic arthritis with hyperammonemia due to *Ureaplasma parvum* infection has never been reported.

## Case presentation

**Fig. 1:**
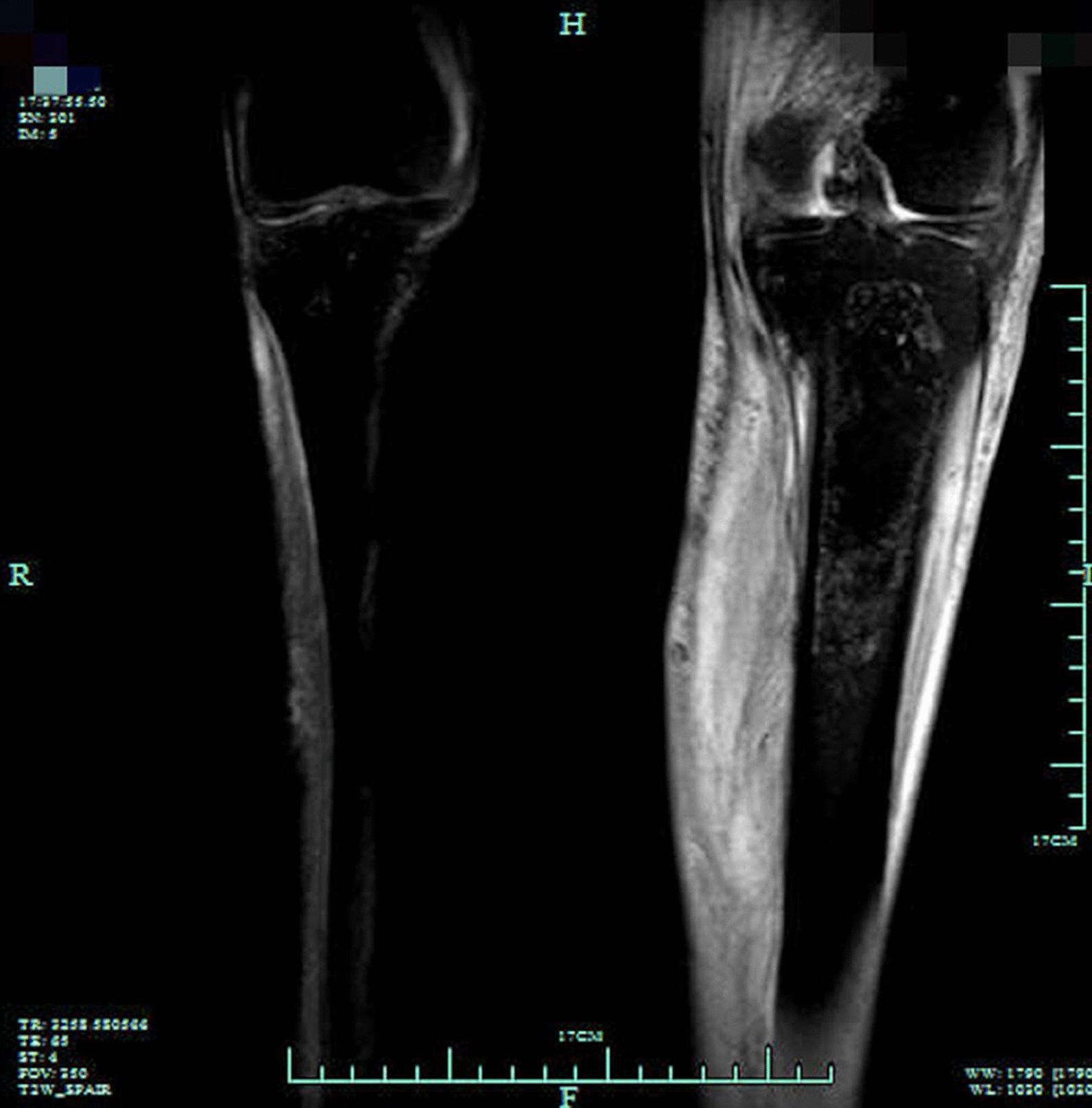
Bilateral knee joint MRI examinations show joint effusion in the left knee, with enhanced T1 signals in the medullary cavity above the tibia, consistent with bone infarct

On June 9th, 2022, A 65-year-old male was admitted due to fever, left lower leg swelling, and pain for more than ten days. His medical history included chronic hepatitis B, alcohol abuse, and tuberculosis with unknown prior treatment. He was initially diagnosed with left leg cellulitis at a local hospital and received antibiotic treatment with cefoperazone-sulbactam and linezolid, but with poor responses. During the presentation to our hospital, his vital signs were temperature 39.9 °C, pulse 118 beats/min, respiration rate 19 times/min, and blood pressure 125/50 mmHg. He was awake but slightly lethargic. Physical examination was unremarkable except that his left lower leg and foot were warm, swollen, and erythematic. No motor or sensory deficit was noted during the neurological examination. The left knee magnetic resonance imaging (MRI) examination reported intra-articular effusion with T1 signal enhancement in the medullary cavity above the left tibia, suggesting bone infarct (Fig. 1). The arthrocentesis showed purulent fluid, and the synovial fluid analysis reported a white blood cell count of 30–50 /HP and a red blood cell count of +/HP. The smear and the Gram staining did not report any pathogen. The synovial fluid was sent for metagenomic sequencing, bacterial and fungal culture, tuberculosis Xpert (a nucleic acid amplification test that uses the GeneXpert Instrument System to diagnose tuberculosis rapidly), and RNA tests. The left leg duplex ultrasound examination showed left lower leg intramuscular calf vein thrombosis. The initial diagnoses were sepsis, left knee septic arthritis, and left lower leg deep vein thrombosis. He received imipenem-cilastatin and linezolid and anticoagulation therapy with heparin. On June 13th, the patient reported melena which was positive for the guaiac test. Meanwhile, he developed delirium, slurry speech, and agitation. The head MRI scan did not show obvious acute large infarcts or hemorrhage. The lumbar puncture was performed with an opening pressure of 250 mmH_2_O, white blood cell count 5*10^6^/L, protein level 0.66 g/L, and glucose 5.2 mmol/L. The serum ammonia level was 292 µmol/L. The liver function tests showed albumin 27.1 g/L, fibrinogen 238 mg/dl, alanine transaminase 27 U/L, aspartate transaminase 46 U/L, and alkaline phosphatase 146 u/L. The coagulation profile reported prothrombin time 14.4 s, partial thromboplastin time 34.5 s (APTT), and international normalized ratio 1.07. The liver sonogram examination revealed a normal uniform hepatic image. The antibiotics were switched to meropenem and linezolid. In addition, lactulose, L-ornithine L-aspartate, mannitol, and hemodialysis therapy were given to lower the ammonia levels. On June 16th, the patient developed a distended abdomen with hypotension. The abdominal X-ray showed an ileus with bowel perforation. Surgery was consulted. However, considering his high risk for surgical operation, conservative treatments were recommended. His repeated laboratory tests showed increased lactate 5.2 mmol/L and ammonia 276 µmol/L. The patient family gave up the treatment and signed out against medical advice.

On June 17th, the synovial fluid metagenomic sequencing test reported 993 sequences of *Ureaplasma parvum* with a relative abundance of 69.3%. Peripheral blood metagenomic assay showed three sequences of *Ureaplasma parvum* with a relative abundance of 5.7%. The tandem mass spectrometry analysis on the blood sample showed ornithine 318.2 µmol/L and glutamate 242.3 µmol/L. The tandem mass spectrometry analysis on the urine sample reported lactate-2 18.9 µmol/L (0–13), 2-hydroxybutyric acid-2 6 µmol/L (0–2), pyruvic acid-OX-2 135.2 µmol/L(0–30), 3-hydroxybutyric acid-2 57.2 µmol/L (0–9), orotic acid-3 µmol/L, and 4-hydroxyphenyllactic acid-2 75.8 µmol/L (0–20). The genetic sequencing did not reveal any potential pathogenic causes of metabolic diseases, including mutations or polymorphisms in genes involved in the urea cycle (*CPS1*, *OTC*, *ASS1*, *ASL*, *ARG1*, *NAGS*, and *SLCA25A15*).

## Discussion and conclusions

Our patient had an initial presentation of left knee septic arthritis but quickly developed into altered mental status. He had poor responses to the antibiotic treatments. Laboratory tests showed hyperammonemia. Finally, the left knee synovial fluid metagenomic sequencing test reported *Ureaplasma parvum*. We consider that the cause of his hyperammonemia was the *Ureaplasma parvum* infection in his left knee joint. Such cases of hyperammonemia due to *Ureaplasma parvum* septic arthritis were never reported. Hyperammonemia is often caused by liver disease or inborn metabolic errors [1]. Ammonia is produced in the intestine by the bacterial degradation of amino acids, amines, purines, and urea. It enters the portal system, where it is broken down during the urea cycle and then removed from the body [4]. Genetic mutations in the enzymes involved in the urea cycle can disrupt the ammonia metabolism and result in hyperammonemia. In our patient, a genetic study did not show any mutations or polymorphisms in the genes involved in the urea cycle, whereas the metagenomic sequencing test suggested a *Ureaplasma parvum* infection in the left knee joint. *Ureaplasma parvum* infection can cause hyperammonemia.

*Ureaplasma parvum* colonizes the genitourinary tract as an opportunistic pathogen [3]. Its terminal structure can induce the host antibody responses. Other virulence factors of *Ureaplasma parvum* include phospholipases A and C, IgA proteases, and urease [5]. The urease in *Ureaplasma parvum* can convert urea into ammonia and carbon dioxide. Clinical studies have shown the relationship between Ureaplasma infection and hyperammonemia [6, 7]. Most cases of Ureplasma-linked hyperammonemia and Ureaplasma septic arthritis were reported in patients with immunocompromised status, such as iatrogenic immunosuppression after organ transplantation or hypogammaglobulinemia from B-cell deficiency [8, 9]. When joints are involved, patients can have reactive arthritis, which is inflammatory arthritis triggered by *Ureaplasma parvum* infection in other body parts. In addition, *Ureaplasma parvum* can invade a joint and cause septic arthritis, even in immunocompetent patients [10–16]. However, none of these cases of septic arthritis reported hyperammonemia in affected patients. The infection from *Ureaplasma parvum* might be underdiagnosed since *Ureaplasma parvum* does not grow in the routine bacterial culture. The development of genetic tests could provide a sensitive and fast diagnosis of the presence of *Ureaplasma parvum*. The treatments for *Ureaplasma parvum* include antibiotics such as fluoroquinolones, doxycycline, clindamycin, and clarithromycin [17]. Commonly used antibiotics, such as beta-lactam and carbapenems, are ineffective against Ureaplasma parvum since these antibiotics inhibit cell wall synthesis by targeting the penicillin-binding protein, whereas *Ureaplasma parvum* lacks a cell wall [18]. In addition, *Ureaplasma parvum* is intrinsically insensitive to linezolid with an extremely high minimum inhibitory concentration [19].

The strength of our study is that we report the first case of septic arthritis with hyperammonemia due to *Ureaplasma parvum* infection. Our study had several limitations. We did not perform the urinalysis on this patient. *Ureaplasma parvum* could cause urinary tract infection, which results in hyperammonemia. We also did not detect the serum ammonia level when this patient was admitted to our hospital. We did not know his baseline serum ammonia level. However, his blood ammonia levels were 292 µmol/L when he had altered mental status, which was significantly higher than the normal values (normal range of ammonia level 9–72 µmol/L). This patient was not likely to have a baseline serum ammonia level close to 300. We considered that the high serum ammonia level resulted from *Ureaplasma parvum* septic arthritis. Finally, we did not perform laboratory tests to examine his immunocompetent status, such as human immunodeficiency virus infection or hypogammaglobulinemia, which might predispose him to the *Ureaplasma parvum* infection. In addition, the diagnosis of *Ureaplasma parvum* was not promptly made for this patient. No effective treatment was applied for *Ureaplasma parvum*, which failed to decrease the ammonia level in this patient. We will pay more attention to these observations in our future clinical practice.

In conclusion, in patients with septic arthritis refractory to the treatments, rare alternative causes, such as infection from Ureaplasma species, should be considered, especially in patients with hyperammonemia.

## Data Availability

The datasets generated and analyzed during the present study are available from the corresponding author upon reasonable request.

## Abbreviation

MRI: Magnetic resonance imaging
